# Spatio-temporal alterations in retinal and choroidal layers in the progression of age-related macular degeneration (AMD) in optical coherence tomography

**DOI:** 10.1038/s41598-021-85110-y

**Published:** 2021-03-11

**Authors:** Wolf-Dieter Vogl, Hrvoje Bogunović, Sebastian M. Waldstein, Sophie Riedl, Ursula Schmidt-Erfurth

**Affiliations:** Department of Ophthalmology, Medical University of Vienna, Vienna, Austria

**Keywords:** Macular degeneration, Image processing

## Abstract

Age-related macular degeneration (AMD) is the predominant cause of vision loss in the elderly with a major impact on ageing societies and healthcare systems. A major challenge in AMD management is the difficulty to determine the disease stage, the highly variable progression speed and the risk of conversion to advanced AMD, where irreversible functional loss occurs. In this study we developed an optical coherence tomography (OCT) imaging based spatio-temporal reference frame to characterize the morphologic progression of intermediate age-related macular degeneration (AMD) and to identify distinctive patterns of conversion to the advanced stages macular neovascularization (MNV) and macular atrophy (MA). We included 10,040 OCT volumes of 518 eyes with intermediate AMD acquired according to a standardized protocol in monthly intervals over two years. Two independent masked retina specialists determined the time of conversion to MNV or MA. All scans were aligned to a common reference frame by intra-patient and inter-patient registration. Automated segmentations of retinal layers and the choroid were computed and en-face maps were transformed into the common reference frame. Population maps were constructed in the subgroups converting to MNV (n=135), MA (n=50) and in non-progressors (n=333). Topographically resolved maps of changes were computed and tested for statistical significant differences. The development over time was analysed by a joint model accounting for longitudinal and right-censoring aspect. Significantly enhanced thinning of the outer nuclear layer (ONL) and retinal pigment epithelium (RPE)–photoreceptorinner segment/outer segment (PR-IS/OS) layers within the central 3 mm and a faster thinning speed preceding conversion was documented for MA progressors. Converters to MNV presented an accelerated thinning of the choroid and appearance changes in the choroid prior to MNV onset. The large-scale automated image analysis allowed us to distinctly assess the progression of morphologic changes in intermediate AMD based on conventional OCT imaging. Distinct topographic and temporal patterns allow to prospectively determine eyes with risk of progression and thereby greatly improving early detection, prevention and development of novel therapeutic strategies.

## Introduction

Age-related macular degeneration (AMD) is a leading cause of vision loss and blindness in the elderly population^1^ with an estimate of 196 million individuals developing AMD in 2020^2^. Individuals with AMD progress through the stages of early, intermediate to late AMD (ie. macular neovascularization (MNV) or macular atrophy (MA)) with a highly variable inter-individual progression speed. It is thought that MA is the invariable endpoint of AMD disease, with MNV developing as a complication of AMD in some individuals^3^. Neovascular AMD is characterized by abnormal blood vessel formation from the choriocapillaris causing exudative changes in the retina. Advanced non-neovascular AMD is characterized by atrophic areas developing in the macula, with loss of photoreceptors, retinal pigment epithelium (RPE) and underlying choriocapillaris. Despite active research in the field, the primary disease mechanisms and individual disease course are not fully understood^4^.

High resolution three-dimensional retinal images acquired by spectral domain OCT (SD-OCT) in combination with automated image analysis enables large-scale *quantitative* analyses of retinal morphology^5^. By including standardized follow-up acquisitions (patho)- morphometric changes can be distinctly assessed over time. However, variance introduced in the acquisition process, such as differing scanning positions of the eye, as well as variations in retinal anatomy need to be tackled when performing longitudinal analysis on a population scale. Therefore, we introduce a normative common reference frame that reduces acquisition-related and anatomic variance by aligning the scans within an individual over time and between individuals by using stable retinal landmarks (optic nerve head (ONH) and fovea). Normative reference frames are widely used in the field of neuroimaging, where individual brains are registered to an atlas or template to obtain spatial normalization^6^. A common approach in such a reference frame is the assessment of significant morphologic changes in the tissue and to distinguish between normal ageing and progressive pathologies such as e.g. Alzheimer’s disease^7^. Obviously, methodologies developed in this field are not limited to the brain and may be transferred to other imaging modalities and organs.

Establishing a common reference frame for OCT images is the initial step towards developing a spatio-temporal atlas detecting the characteristic pathomorphologic changes and the pathognomonic disease course in AMD. Such an atlas may ultimately provide a normative database of retinal ageing and age-related retinal pathology, allowing to compare the retinal configuration and progression speed of an individual to the general population - similarly to normative databases widely available for the evaluation of retinal changes in glaucoma^8^, but currently lacking for the much more frequent AMD disease. In this study we used the common reference frame to assess the typical morphologic distribution and temporal development of neurosensory, RPE and choroidal layer structures in a well phenotyped cohort of patients with intermediate AMD undergoing continuous monitoring using SD-OCT. In particular, we focused on the onset of advanced AMD and assessed the spatio-temporal development distinguishing patients close to progression followed by conversion from patients that remained in an earlier stage of AMD. Our results identified characteristic changes in retinal structures when developing MA, as well as choroidal changes indicating conversion to MNV. This work may be seen as an essential step in understanding disease pathways and in assessing individual risk estimates in a disease of epidemic proportions with high individual variability such as AMD.

## Methods

### Population and definitions

This study was a post-hoc analysis of SD-OCT-imaging data collected during the HARBOR clinical trial^9^. The study design and primary outcomes have been published in detail previously^10,11^. HARBOR included patients with neovascular AMD and evaluated efficacy and safety of intravitreal ranibizumab for two doses (0.5 and 2.0) and two regimens (monthly, as needed after 3 loading doses) in 1,097 patients of age $\ge 50$ monitored in a standardized monthly schedule for 24 months. At each visit a Zeiss Cirrus SD-OCT of both eyes was acquired. The inclusion criteria for HARBOR were treatment-naïve MNV lesions with a classic MNV component, or occult MNV in the study eye. No limiting criteria regarding the condition of the fellow eye were predefined.

All study procedures were conducted in accordance with the tenets set forth in the Declaration of Helsinki and following Good Clinical Practice guidelines. All patients provided written informed consent before enrollment into the clinical trial. For the retrospective analysis of the image data, approval was obtained by the Ethics Committee at the Medical University of Vienna, Austria.

**Diagnostic criteria and adjudication** In our study we analysed the fellow eye SD-OCT images of patients presenting with intermediate AMD at baseline. Two masked retina specialists independently identified the time of conversion to advanced AMD, i.e. MNV or MA. Mandatory criteria for MA were: Presence of choroidal hypertransmission in combination with loss of photoreceptors and RPE, excluding scrolled RPE layer areas indicative of a RPE tear^12^. The onset of MNV was diagnosed based on the presence of intra- and/or subretinal fluid with fibrovascular pigment epithelial detachment (PED) and/or subretinal hyperreflective material (SHRM)^13^. Cases with disagreement were adjudicated for final diagnosis by a senior retina specialist. Patients without signs of AMD, i.e. presenting no drusen, or having undergone AMD treatments previously were excluded from the study.

### Quantitative OCT measures in a common reference frame

**Figure 1:**
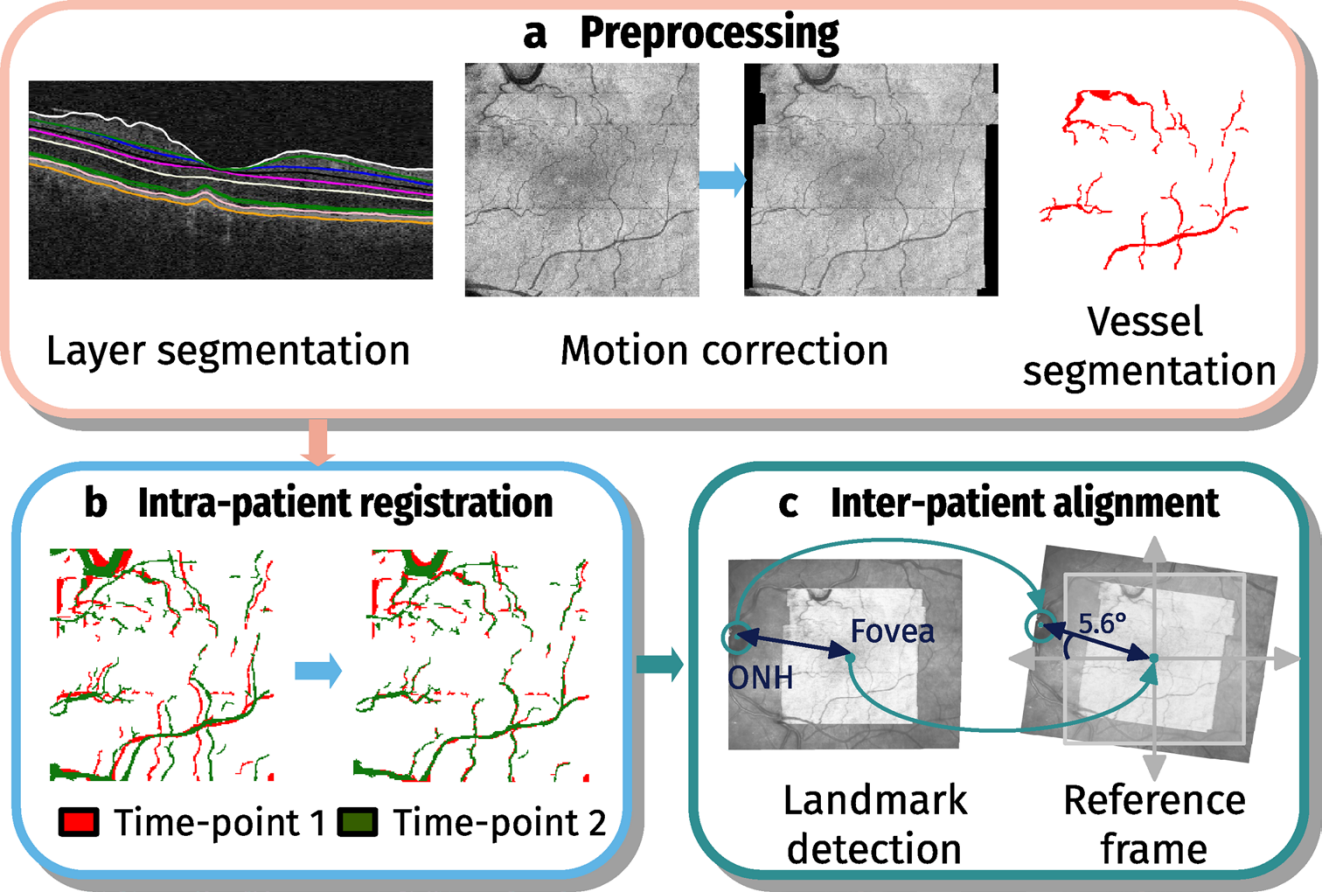
Steps to generate a reference frame by intra-patient and inter-patient alignment. (**a**) Preprocessing steps containing layer segmentation, correction of motion errors and segmentation of main vessels. (**b**) Intra-patient registration by aligning the vessel structure. (**c**) Inter-patient registration comprising optic nerve head (ONH) and fovea landmark detection in the SLO and SD-OCT image. The fovea position is moved to the centre of reference frame and the OCT is rotated such that the ONH centre is at the population mean position.

To obtain a spatio-temporal normative atlas we propose a two step intra-patient and inter-patient registration process, using the vessel structure for intra-patient alignment and fovea center / ONH centre line to obtain correspondences between patients (Fig. 1). This method is a consequent continuation of our previous work^14^.

We performed the following steps in the registration pipeline: First, we reduced motion artifacts occurring during acquisition caused by eye movements using the method described by Montuoro et al.^15^. Then, we generated the vessel probability map from the en-face optical coherence tomography (OCT) projection by using for each A-scan the 0.7 percentile intensity value within a range of ${30} \,\upmu \text{m}$ above the RPE border, in case of a large vessel shadow is cast below, causing a lower intensity in the underlying RPE area. The percentile image was first resampled to isotropic pixels, followed by denoising using non-local means with H = 0.025 and afterwards a histogram normalization using contrast limited adaptive histogram equalization (CLAHE)^16^ with a clip-limit of 0.4. By applying a vesselness filter^17^ we obtained the vessel probability map, ${\mathscr {P}}^{ vessel }$. In addition, we converted ${\mathscr {P}}^{ vessel }$ to a binary vessel map, ${\mathscr {P}}^{ binary }$ using a threshold of 0.4 and removing objects smaller than 80 pixels. The intrapatient registration was computed with software package ANTs^18^ (Version 2.3.1). First, we applied a rigid registration followed by an affine registration using a four-level Gaussian pyramid. As similarity metric we used windowed normalized cross-correlation with a window size of 9 for ${\mathscr {P}}^{ vessel }$ and mean-squares error for ${\mathscr {P}}^{ binary }$. The first acquisition in the image series were used as reference to which all scans were registered to. In early/intermediate AMD images this scan is commonly affected least by the pathology.

For interpatient registration we applied an automated fovea position detection algorithm to precisely locate the center of the fovea. The centroid of the minimum thickness between inner limiting membrane (ILM) and ganglion cell layer (GCL) indicated the center of the foveal depression. Neurosensory layers were identified using the Iowa reference algorithm (Retinal Image Analysis Lab, Iowa Institute for Biomedical Imaging, Iowa City, IA)^19^. The second reference point was the ONH center, which is usually not visible in fovea-centered OCT images. Thus, we detected the circular shape of the ONH structure in the corresponding scanning laser ophthalmoscope (SLO) image using a RANSAC based circle detection^20^ on a binary version of the image obtained by applying an adaptive threshold filter. The center of the detected circle was determined as ONH center. All positions were manually verified. Interpatient alignment was performed in a rigid way such that the fovea centre was at the centre of the reference frame and the angle between fovea and the centre of the optic disc versus the horizon was consistent with the population mean of $5{.}6^\circ$^21^. Scans of right eyes were mirrored. For the reference frame we defined a pixel resolution of $15\times 15 \, \upmu \hbox {m}$ and an image size of $512\times 512$ pixels ($=7{.}68 \times 7.68 \hbox {mm}$).

**Pathognomonic features in the common reference frame ** We obtained neurosensory, RPE and choroidal layer structures by automated layer segmentation using IOWA reference algorithms^19,22^ and a modified version with differing smoothness constraints to segment RPE in the presence of drusenoid deposits^23^. Retinal thickness maps were obtained by computing for each A-scan the Euclidean distance between two segmented layers in $\upmu \hbox {m}$ units.

**Figure 2:**
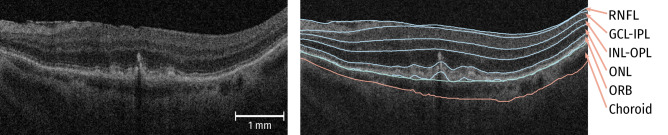
Example of automatic layer segmentation. 6 retinal layers and the choroid are segmented. *Retinal nerve fibre layer (RNFL), ganglion cell layer (GCL), inner plexiform layer (IPL), inner nuclear layer (INL), outer plexiform layer (OPL), outer nuclear layer (ONL), and outer retinal hyperreflective bands (ORB) comprising retinal pigment epithelium and outer photoreceptor segments.*

We included maps for 1) retinal nerve fibre layer (RNFL), 2) GCL + inner plexiform layer (IPL), 3) inner nuclear layer (INL) + outer plexiform layer (OPL), 4) outer nuclear layer (ONL), and 5) outer retinal hyperreflective bands (ORB) comprising RPE and outer photoreceptor segments (Fig. 2). Choroidal thickness was defined as the distance between Bruch’s membrane (BM) and the choroidal posterior boundary. Due to low signal level in the choroid, an exact segmentation is a challenging and error-prone task. Hence, we applied in addition a texture analysis approach, in the sense of radiomics used in radiographic medical images^24^, to obtain a more robust characterization of choroidal changes. We computed gray-level co-occurence matrix (GLCM)and the six main Haralick texture descriptors^25^ for the choroidal area provided by the software framework National Library of Medicine Insight Segmentation and Registration Toolkit (ITK)^26^. The computed texture descriptors were Energy, Entropy, Inverse Difference Moment, Inertia, Cluster Shade and Cluster Prominence. In detail, we extracted for each A-scan a ROI of 5 pixel width and 300  $\upmu \hbox {m}$ depth starting at BM. For each patch we computed GLCM with 16 bins using an intensity range of 0 to 128. To obtain rotation invariance we computed GLCMs for pixel pairs in nasal/temporal (horizontal), inferior/superior (vertical) and diagonal directions. Considering the anisotropy of the input image spacing, we chose as distance of 6 pixels and 1 pixel in inferior/superior and nasal/temporal direction, respectively. Haralick texture features^25^ were computed for all GLCMs and averaged afterwards. We computed texture descriptors using “ScalarImageToTextureFeaturesFilter” from the ITK framework^26^ (Version 5.0.0).

### Statistical analyses

According to the diagnostic grading described above we divided the HARBOR fellow-eye dataset into three groups: eyes, which progressed to MNV or MA within the study period of two years, and eyes that did not show any clinical sign of conversion.

For a comprehensive spatial or topographic analysis, we computed for each feature a cross-sectional mean and standard deviation (std) map per group across all eyes, as well as the difference of means between groups. Scans from 1 month prior conversion (MNV and MA) and last available scan in study (non-progressor) were chosen. At each spatial location the group means were compared for significant difference (i.e. due to a thinning or thickening of layers) using a Mann-Whitney U test. P-values were adjusted for multiple testing by controlling the false discovery rate (FDR) of 5% using the procedure of Benjamini-Hochberg^27^. Furthermore, to obtain an easier interpretable summary of the topography, we computed and plotted the mean feature thickness as a function of eccentricity, denoted in the following as *topographic profiles*. For each of these profiles Mann-Whitney U tests were used to determine significant differences in the distribution of the groups, and p-values were also corrected for FDR.

Longitudinal analysis of retinal morphology were performed on computed feature mean values within the central foveal area of 3 mm diameter. Smoothed mean trajectories of features were plotted using locally estimated scatterplot smoothing (LOESS) fits. We used a longitudinal mixed effects model to perform univariate analysis for each feature. This approach enables to identify significant differences in mean at baseline and in the slope of the mean trajectories. To account for right-censoring in the data, we used a proportional hazards survival approach, which we combined with the mixed effects model into a joint model^28^. We modeled development of features over time as a linear growth curve mixed effects model with intercept and slope as fixed and random factors. Groups were included as categorical covariates with dummy coding and non-progressors as reference. For inference, we used a Bayesian MCMC approach provided by the R package ’JMBayes’^29^ (Version 0.8.83) with ’JAGS’ Gibbs sampling^30^ (Version 4.3.0). We used 900 iterations for MCMC sampling with 600 iterations for burn-in and 300 for thinning.

## Results

### Patient population

518 fellow eyes of 1,097 patients included in the HARBOR trial were diagnosed with intermediate AMD at baseline. 50 and 135 eyes developed MA or MNV during the 24-month follow-up period, respectively. 333 eyes did not exhibit any significant progression to advanced AMD. Demographic characteristics of the cohort are given in Table 1. Overall, 12,135 SD-OCT scans were transformed into the reference frame, from which 10,040 scans were used for further analysis. Scans were excluded when layer segmentation failed due to bad image quality and no valid en-face projection for vessel detection could be computed, or where motion artefacts prevented intrapatient registration. The correctness of registration was verified in all cases by overlaying the registered en-face projections and by validating the correct positions of fovea and ONH centers.

**Table 1:** Patient characteristics.

Group	#	Age mean ± std	Female # (%)
MNV	135	78.5 ± 8.5	94 (69.6)
MA	50	81.2 ± 6.4	29 (58.0)
non-progressor	333	77.4 ± 8.2	186(55.9)
all	518	78.1 ± 8.2	309 (59.7)

### Characteristics of retinal layer thicknesses and choroid

**Figure 3:**
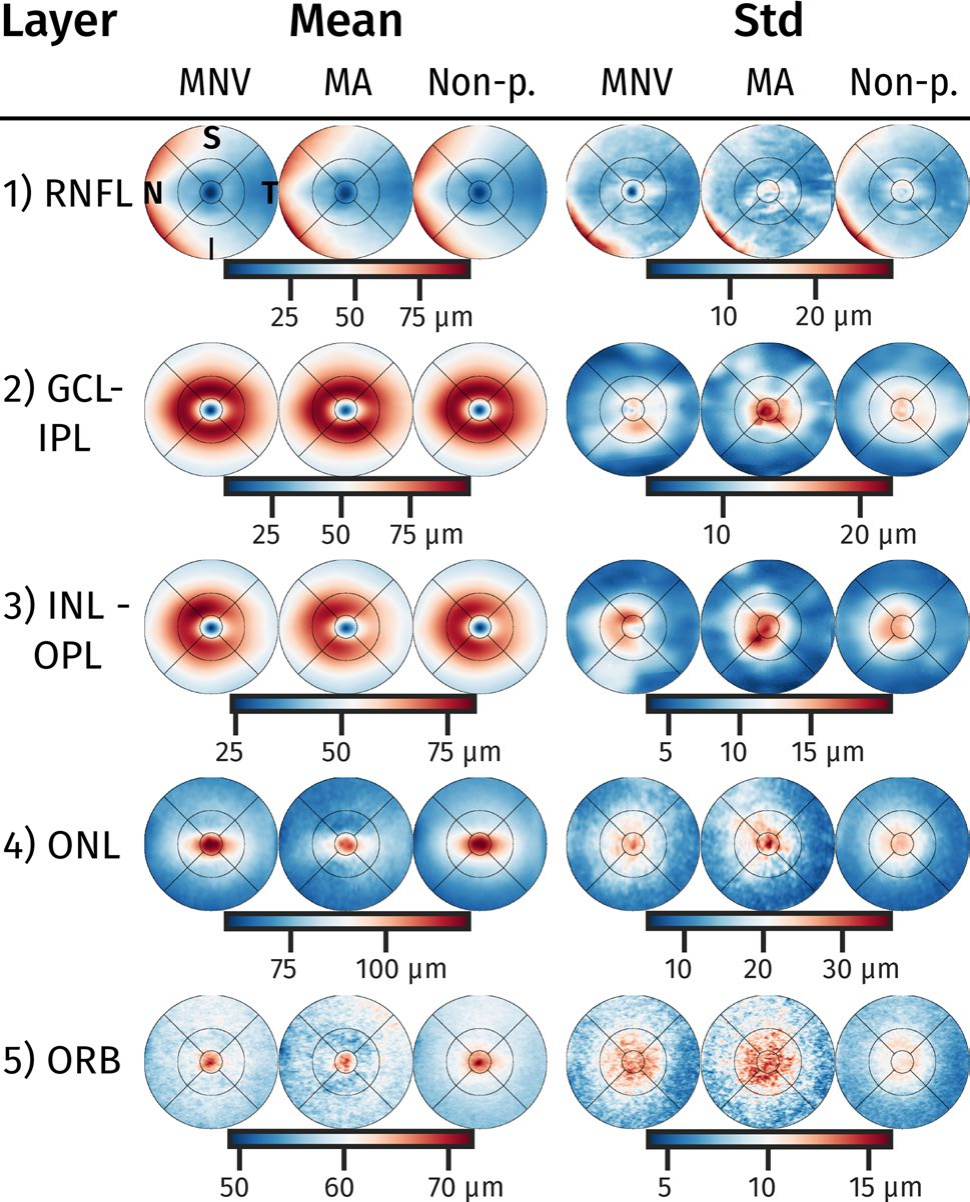
Mean and standard deviation (std) thickness maps of layers. Population mean/std maps within the reference space for the three groups macular neovascularization (MNV), macular atrophy (MA) and non-progressors (Non-p.). For orientation a standard grid with 1,3 and 6mm diameter centred at fovea is shown. *Retinal nerve fibre layer (RNFL), ganglion cell layer (GCL), inner plexiform layer (IPL), inner nuclear layer (INL), outer plexiform layer (OPL), outer nuclear layer (ONL), and outer retinal hyperreflective bands (ORB). N (Nasal), S (Superior), T (Temporal), and I (Inferior).*

**Figure 4:**
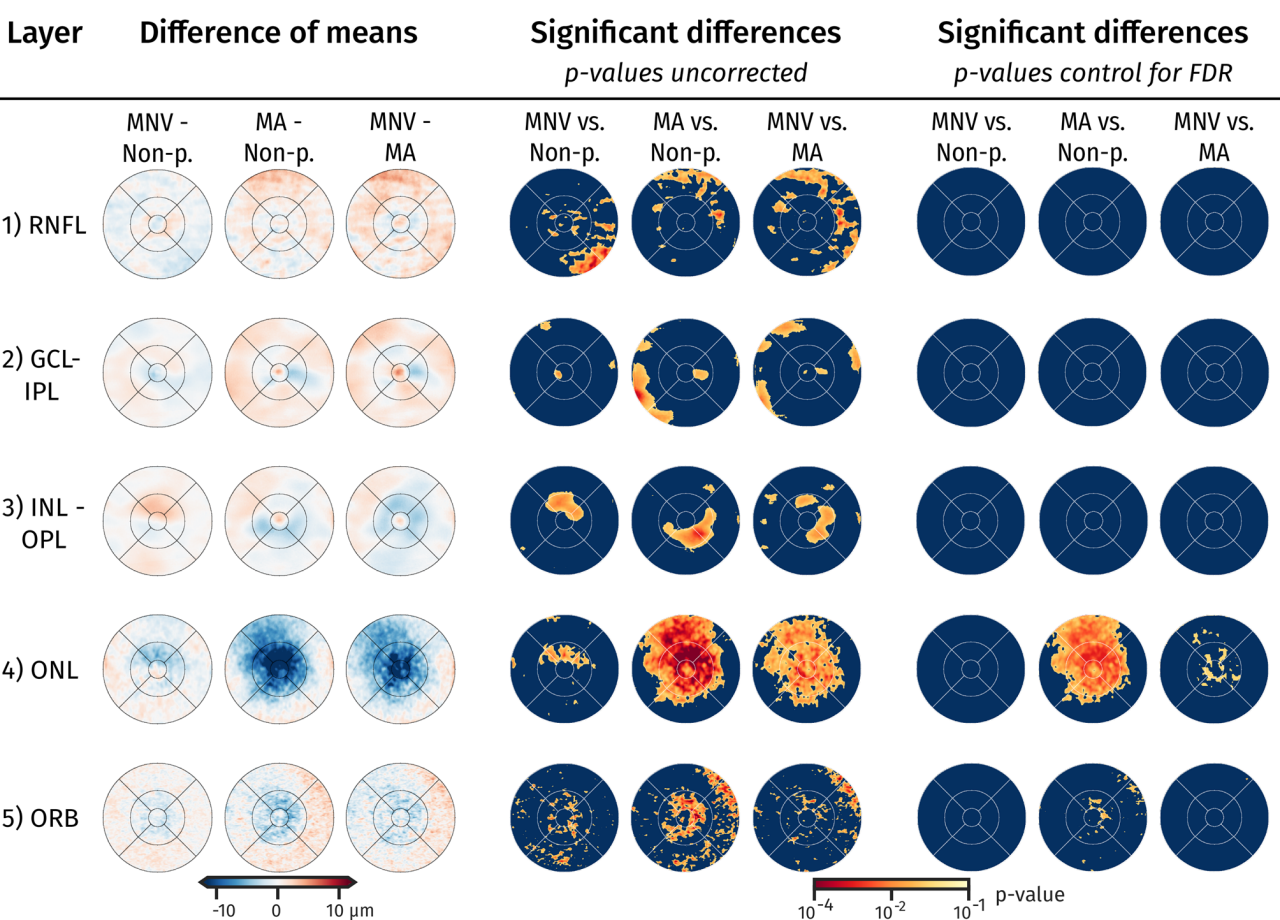
Difference of mean for retinal layers. We show the difference of the mean maps for the specific layers (*left*), and the statistic test-result whether the distribution of groups are significant different tested at each anatomic location (*centre*). P-values of a Mann-Whitney U test are shown. On the *right* are the p-values corrected for 5% false discovery rate. *Retinal nerve fibre layer (RNFL), ganglion cell layer (GCL), inner plexiform layer (IPL), inner nuclear layer (INL), outer plexiform layer (OPL), outer nuclear layer (ONL), and outer retinal hyperreflective bands (ORB)*.

**Figure 5:**
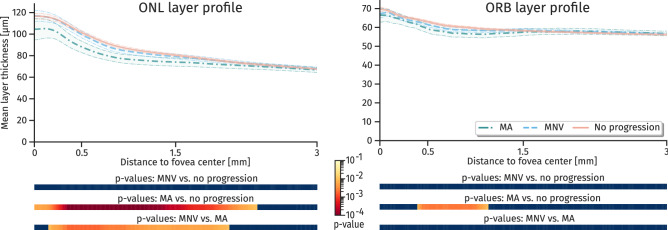
Topographic profile of layers outer nuclear layer (ONL) and outer retinal hyperreflective bands (ORB). The area around mean curves show the 0.95 confidence interval of means. The bars below the profiles illustrate the significance level in terms of p-values from a Mann-Whitney U test at the given distance to fovea. P-values are corrected for 5% false discovery rate.

Figure 3 shows the mean and standard deviation of the layer thickness development for the three groups MNV, MA and non-progressors (Non-p). The MA population consistently demonstrated a thinner ONL layer, and in general a higher variance in GCL-IPL, INL-OPL and ORB within the 1.5 mm eccentricity. The difference of means thickness (Fig. 4) showed a significant thinner ONL and ORB for subjects progressing to MA in non-foveal areas. For the other layers, significance in mean difference was not reached after correcting for multiple testing. The topographic profile (Fig. 5) confirmed a significant thinning of the ONL from 0.3 to 2 mm eccentricity with increased thinning closer to the fovea. For the ORB, the thinning was significant within an eccentricity of 0.4 to 1 mm. Other topographic profiles did not exhibit significant differences between the groups (Supplementary Figure S1).

**Figure 6:**
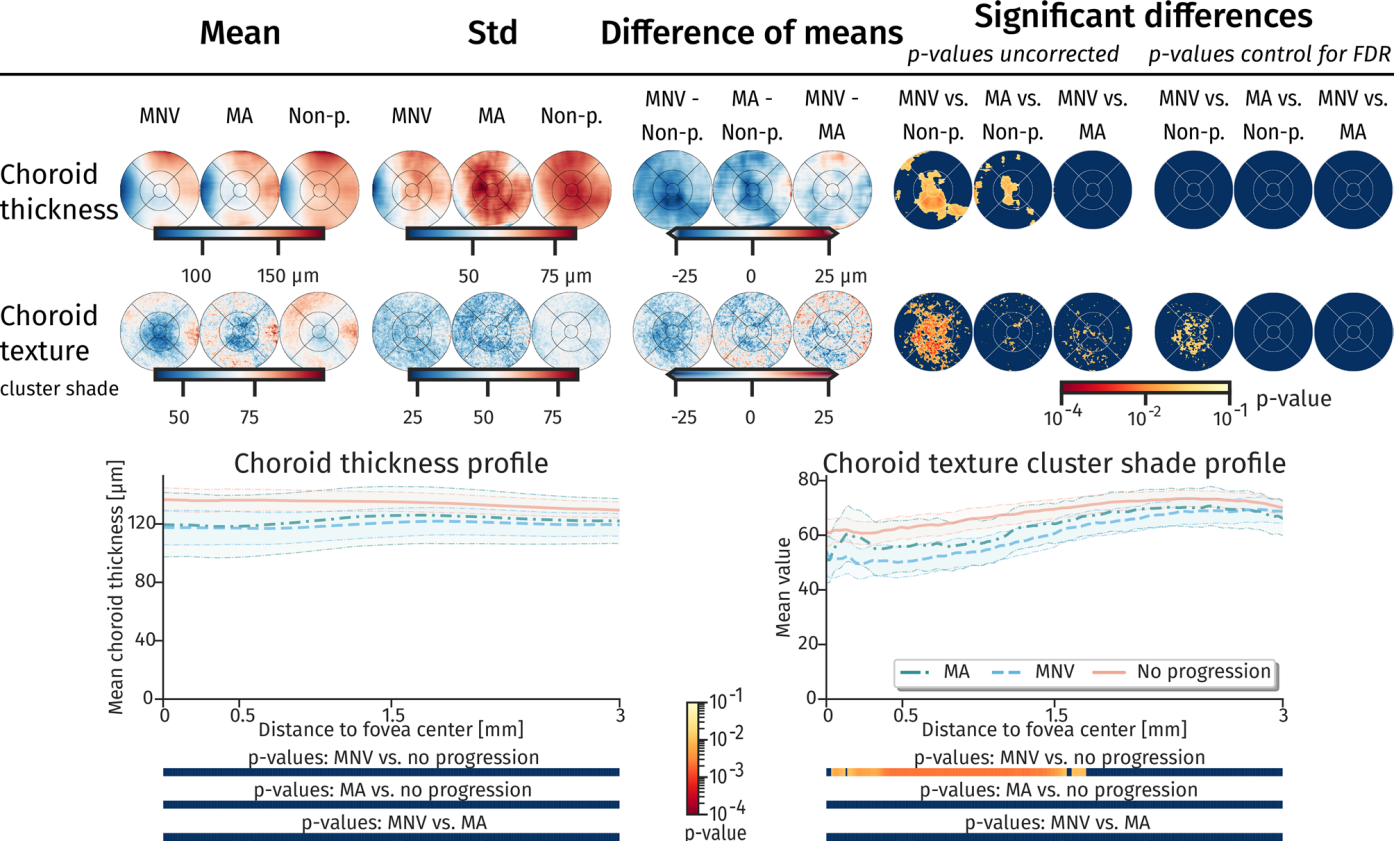
Topographic maps and profiles of choroidal thickness and texture. Spatial distribution (top) and topographic profile (bottom) of choroidal thickness and choroidal texture property (cluster shade), which shows significant differences between groups macular neovascularization (MNV), macular atrophy (MA) and non-progressor (Non-p). Confidence interval and p-value properties are identical to Figs. 3 and 4.

For choroidal thickness, a distinctly thinner layer could be observed for MNV patients (Fig. 6). After correcting for FDR the difference was not statistically significant in the difference of mean maps and in the topographic profile. Textural characteristics that is less sensitive to segmentation errors, showed, however, significantly different values for the texture property ‘cluster shade’ and ‘cluster prominence’, which was also significant in the 0.5 to 1.5 eccentricity both in topographic maps and profiles (Fig. 6, Supplementary Figure S2).

**Figure 7:**
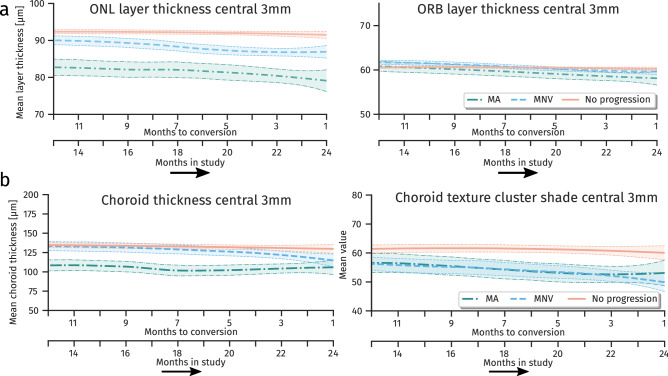
Longitudinal development of retinal and choroidal layer mean thickness and choroidal texture properties. (**a**) Mean thickness values over time for layers outer nuclear layer (ONL) and outer retinal hyperreflective bands (ORB) in groups macular neovascularization (MNV), macular atrophy (MA) and non-progressors. (**b**) Mean thickness and texture descriptor values over time for the choroid. Curves show the local mean thickness with respect to time to conversion for MNV and MA, and with respect to time in study for non-progressor. A local and smooth mean is obtained by LOESS. The interval shows the 0.95 confidence of LOESS fit.

Results of longitudinal modeling are subsumed in Table 2. In brief, in the central 3 mm eccentricity compared to the non-progressor group both MNV and MA progressors showed a thinner ONL (MNV: $p<0{.}001$, MA: $p<0{.}001$) and a faster thinning over time (MNV: $p=0{.}004$, MA: $p=0{.}022$), which was more pronounced for MA conversion (Fig. 7A). A faster thinning was also observed for both groups in the ORB (MNV: $p < 0{.}001$, MA: $p < 0{.}001$), resulting in a significant thinner ORB at one month before conversion. An overall significant thinning of choroid could be observed ($p<0{.}001$). No significant differences in choroid thickness and changes were detected for progressors. Notwithstanding, significant differences in choroid texture descriptors could be observed already at baseline (MNV: $p=0{.}022$, MA: $p=0{.}034$) (Fig. 7B).

**Table 2:** Regression coefficients of longitudinal joint models. Regression coefficients for ONL, ORB and choroidal thickness, as well as choroidal texture showing the baseline thickness or texture value and change over time per month for the three groups. Non-progressors were used as reference and values for progressors are relative to non-progressor values. P-values report the statistical significant difference of the value to 0. Bold values indicate statistical significance ($\hbox {P}< {0.05}$).

Regression coefficient	Mean value (0.95 CI)	P-value	Mean value (0.95 CI)	P-value
	**ONL thickness** [$\mu m$]		**ORB thickness** [$\mu m$]	
Non-p. baseline thickness	90.78 (89.47/92.05)		60.68 (60.16/61.18)	
MNV relative baseline thickness	$\mathbf {{-}3.1}$ ($-$ 5.41/$-$ 0.92)	0.008	0.31 ($-$ 0.68/1.29)	0.534
MA relative baseline thickness	$\mathbf {{-}9.15}$ ($-$ 12.64/$-$ 5.47)	$<{0.001}$	$-$ 1.15 ($-$ 2.56/0.22)	0.114
Non-p. thickness change per month	$\mathbf {{-}0.11}$ ($-$ 0.13/$-$ 0.084)	$<{0.001}$	$\mathbf {{-}0. 023}$ ($-$ 0.036/$-$0.010)	0.002
MNV relative change per month	$\mathbf {{-}0.080}$ ($-$ 0.13/$-$ 0.022)	0.004	$\mathbf {{-}0.094}$ ($-$ 0.13/$-$ 0.064)	$< {0.001}$
MA relative change per month	$\mathbf {{-}0.11}$ ($-$ 0.20/$-$ 0.013)	0.022	$\mathbf {{-}0.12}$($-$ 0.17/$-$ 0.070)	$< {0.001}$
	**Choroid thickness** [$\mu m$]		**Choroid texture (cluster shade)**	
Non-p. baseline value	132.19 (126.17/138.48)		69.06 (65.66/72.49)	
MNV relative baseline value	4.84 ($-$ 5.95/16.40)	0.392	$\mathbf {{-}7.82}$ ($-$ 14.47/$-$1.38)	0.022
MA relative baseline value	$-$ 3.31 ($-$ 16.48/10.53)	0.626	$\mathbf {{-}9.65}$ ($-$ 18.18/$-$0.77)	0.034
Non-p. change per month	$\mathbf {{-}0\cdot 47}$ ($-$ 0.62/$-$0.33)	$< {0.001}$	$\mathbf {{-}0.24}$ ($-$ 0.33/$-$0.14)	$<{0.001}$
MNV relative change per month	$-$ 0.38 ($-$ 0.74/0.0074)	0.066	0.086 ($-$ 0.16/0.34)	0.454
MA relative change per month	$-$ 0.20 ($-$ 0.80 /0.41)	0.516	0.16 (0.21 /$-$ 0.26)	0.440

*Non-progressor (Non-p.), Macular neovascularization (MNV), macular atrophy (MA), confidence interval (CI)*.

## Discussion

The purpose of our study was to establish a reference frame for spatio-temporal analysis of macular OCT data allowing population-level study of topographic and longitudinal features of disease. Applying the newly developed framework on a large-scale clinical dataset using automated processing, we successfully identified morphologic markers characteristically associated with and potentially predicting progression from intermediate to advanced AMD, one of the leading eye diseases of modern times.

In detail, we established a common reference frame to build an exemplary spatio-temporal macular OCT atlas in an AMD population. It allowed us to identify spatio-temporal relationships of changes in anatomical regions independent of variances introduced by individual anatomy, disease progression and acquisition factors. We assessed the association of AMD progression with morphologic changes in the retina on a population level, both for local anatomic regions, i.e. spatially and over time, i.e. temporally. By processing data from intermediate AMD patients progressing to advanced stage disease, we were able to detect a significant thinning of the neurosensory retinal elements, i.e. the ONL within 2 mm eccentricity and ORB within 0.4 to 1 mm eccentricity for patients converting to MA. In contrast, choroidal changes were found in the central 1.5 mm eccentricity for patients with imminent onset of MNV. Considering the longitudinal aspect, a faster thinning speed of ONL and ORB towards the point of conversion of MNV or MA, as well as a faster choroidal thinning for MNV progressors was identified. Such pathognomonic processes are of major relevance for understanding the pathomechanisms of conversion and may serve as biomarkers for risk assessment and early detection of conversion in a screening setting.

Comparison of progressors versus non-progressors within the reference frame revealed an association between thinning of ONL and ORB with progression to MA. This confirms recent findings, where a significant thinning of ONL and photoreceptor-inner segment/outer segment (PR-IS/OS) in the central circle (0.5 mm eccentricity) and/or inner circles (0.5-to 1.5 mm eccentricity) was reported^31^. Considering the characteristics over time, an increase in ORB and ONL thinning may be a sign of consecutive progression to MA. This confirms a recent study^4^, where ONL and ORB thickness as well as changes in ORB morphometry were identified as predictive factors for MA progression. The thinning of ONL may be partially explained by an enlargement of the RPE-BM complex due to extracellular accumulation of drusen material eventually causing a compression of ONL. However, MA progressors showed an even thinner ONL compared to MNV progressors, even though drusen were equally present in both groups^32^. Whereas loss of photoreceptors and thinning of ONL in combination with degradation of RPE are well-known signs of atrophic areas^33,34^, it is interesting to see that these processes occur already much earlier in the disease course and long before the onset of late-stage AMD. Furthermore, the speed of degradation increases when approaching the time of conversion. It has to be mentioned that a clear distinction between ONL and Henle fiber layer is not possible in standard SD-OCT images^35^. Thus, specific changes in photoreceptor nuclei located within ONL and photoreceptor axons and Mueller cells located in the HFL could not be individually assessed in this study. We also observed changes in other layers, i.e. in the INL-OPL within 0.5 to 1.5 eccentricity (Fig. 5 and Supplementary Figure S1) for MA progressors. However, statistical significance was not achieved after correction for FDR. Still, this might be an indication of characteristics of a so-called nascent atrophy that is characterized amongst others by a diminishment of the OPL and INL^12^. Whereas a significant GCL-IPL thinning was reported for patients with intermediate AMD compared to healthy control^36,37^, we observed no significant thinning within patients consecutive to progression versus non-progressors. This indicates that there are alterations in the innermost retinal layers, though they do not provide an indication of progression within intermediate AMD stage.

Measuring choroidal properties in standard SD-OCT imaging is a challenging task, as the signal to noise ratio (SNR) rapidly drops below the RPE layer due to absorption of the incident light by the dark pigment. Thus, we additionally introduced texture descriptors for the choroidal area, which do not depend on an exact segmentation of the choroid. However, interpretability of texture features is limited due to missing reference to a physical scale, as for instance thickness values. The feature “cluster shade” measures the uniformity of the underlying texture, with lower values suggesting higher uniformity. In the MNV progressors, we observed a significantly lower value in the 0.5 to 1.5 eccentricity. A lower value may indicate larger uniform areas, which are in this case often areas without signal and thus without choroidal vasculature. This may be an indication of thinning in the choroidal vasculature. Novel imaging techniques, such as swept-source OCT and OCT angiography provide better SNR in the choroid or more details about vascular patterns. Using these modalities, changes in the choroid specific for patients developing MNV could be observed earlier^38^. However, despite the limitations, we were able to detect significant effects in the choroid for patients progressing to MNV in terms of choroidal thinning and in textural differences. These findings are consistent with recent publications suggesting choroidal involvement for predicting progression in a deep learning model^39^. In our population, we could not identify a statistical significantly thinning of the choroid for MA progressors. However, other studies identified choroidal thinning as a risk factor^40^.

To facilitate current and future endeavours in the retinal OCT analysis field, we introduced a common reference frame for spatial and temporal statistical analysis on a population level. The en-face maps and corresponding statistical tests allow to assess topographic properties and morphometric differences in specific patient groups in a normative environment on a considerably finer scale than using the grids typically applied in retinal studies. The topographic profiles, on the other hand, provide a different view on the data, where the property of having an exact spatial location is sacrificed for obtaining a representation that is easier to interpret and invariant to anatomical direction (e.g. nasal vs. temporal). This approach allows assessment of small effects that are not correlated with anatomical directions, except for distance to fovea. A higher sensitivity can be observed for instance in the ORB, where the significantly different area was larger in the topographic profile than in the en-face map. These are important insights in the advancing field of morphological analyses of disease progression. The reference frame may also be applied to other study cohorts such as healthy versus various stages of AMD (mild, moderate, severe)^31,41^ to obtain a broader view on disease progression. Furthermore, with the rapid development in AI and particular deep learning, more accurate and fine-grained segmentations will become available^5^, such as precise RPE and photoreceptor segmentations^42,43^, which may lead to novel findings and highlight different aspects of disease progression when analysing these in the proposed reference frame. A spatio-temporal atlas may also incorporate other OCT modalities such as adaptive optics OCT (AO-OCT), OCT angiography (OCT-A) or directional OCT (D-OCT)^35^ that provide complementary information regarding the disease course.

**Limitations.** First, in our study we included participants of the HARBOR clinical trial of neovascular AMD in one eye and intermediate AMD in the fellow eye. The risk for developing MNV is enriched in this cohort. This allows us to study MNV conversion in more detail due to larger sample size. However, it is unknown how the findings generalize to a more general AMD population with unilateral or bilateral early/intermediate AMD. Second, in this study we compared only patients with early/intermediate AMD and did not provide a healthy age-matched population that would allow to identify pathognomonic changes already for early stages of AMD^41^ and contrast normal ageing versus pathological progression. Third, the number of MA progressors was rather small. A larger cohort might reveal additional significantly different retinal areas or more subtle changes between the groups. All mentioned limitations show that there is a vital necessity in early and intermediate AMD studies with regular follow-up OCT acquisitions over a longer period.

In conclusion, we present an innovative framework based on standard OCT imaging that allows to assess spatio-temporal morphologic changes in the retina on a population level as well as for individuals. We analysed retinal and subretinal layer changes in patients with intermediate AMD that allowed us to assess the time course of pathomorphology and in particular the different pathways in AMD conversion towards MNV and MA. Progressors to MA are in particular characterized by significant changes in the ONL layer thickness, whereas for MNV progressors there is an indication of choroidal changes before onset to late-stage AMD. This work is an initial step towards a comprehensive atlas of retinal changes in the ageing human eye to obtain a deeper understanding of underlying disease mechanisms and disease course. The proposed normative spatio-temporal atlas and resulting findings may lead to a more precise and personalized risk assessment in age-related macular disease. Advanced analysis tools based on deep learning will widely open our insight into subclinical morphological pathology in a qualitative and quantitative manner and offer reliable, non-invasive and precise methods for personalized screening and disease monitoring.

## Supplementary information

Supplementary material 1 (pdf 5001 KB)

## Abbreviations

AMD: Age-related macular degeneration
AO-OCT: Adaptive optics OCT
BM: Bruch’s membrane
D-OCT: Directional OCT
DOM: Difference of means
FDR: False discovery rate
GCL: Ganglion cell layer
GLCM: Gray-level co-occurence matrix
HFL: Henle fiber layer
ILM: Inner limiting membrane
INL: Inner nuclear layer
IPL: Inner plexiform layer
LOESS: Locally estimated scatterplot smoothing
MA: Macular atrophy
MNV: Macular neovascularization
OCT: Optical coherence tomography
OCT-A: OCT angiography
ONH: Optic nerve head
ONL: Outer nuclear layer
OPL: Outer plexiform layer
ORB: Outer retinal hyperreflective bands
PED: Pigment epithelial detachment
RNFL: Retinal nerve fibre layer
RPE: Retinal pigment epithelium
SD-OCT: Spectral domain OCT
SHRM: Subretinal hyperreflective material
SLO: Scanning laser ophthalmoscope
SNR: Signal to noise ratio
std: Standard deviation
