# Supplementary material for: Spatio-temporal alterations in retinal and choroidal layers in the progression of age-related macular degeneration (AMD) in optical coherence tomography
Source: Sci Rep. 2021 Mar 11;11:5743. doi: 10.1038/s41598-021-85110-y (PMC7952738; doi:10.1038/s41598-021-85110-y)
Supplement: Supplementary file 1 — Supplementary material 1 (pdf 5001 KB) [file 41598_2021_85110_MOESM1_ESM.pdf]

Spatio-temporal Alterations In Retinal And Choroidal Layers In the Progression of Age-related Macular Degeneration (AMD) in Optical Coherence Tomography

Wolf-Dieter Vogl[1], Hrvoje Bogunović[1], Sebastian M. Waldstein[1], Sophie Riedl[1] and Ursula Schmidt-Erfurth[1,*]

[1]Department of Ophthalmology, Medical University of Vienna, Austria
[*]ursula.schmidt-erfurth@meduniwien.ac.at

Supplementary Figures

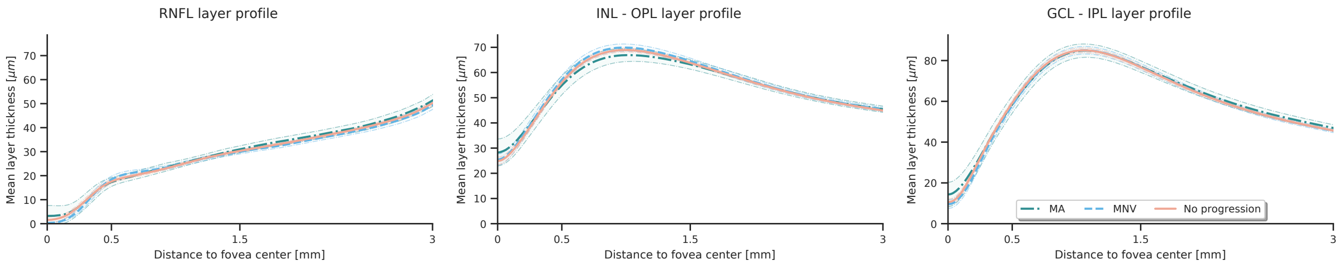

Supplementary Figure S1. Topographic profile of inner retinal layers. No significant difference between the groups can be observed.

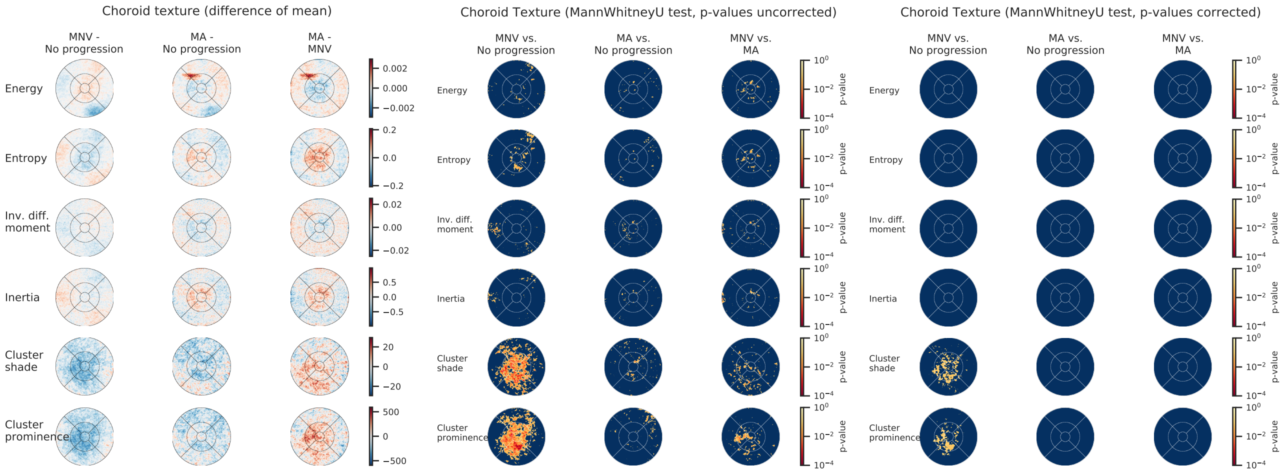

Supplementary Figure S2. Choroidal texture features. Difference of mean in choroidal texture features (left). Hypothesis test of significant difference in mean using Mann-Whitney-U (center) and with FDR correction for multiple testing using Benjamini-Hochberg (right).
